# Mechanisms Involved in Alleviation of Intestinal Inflammation by Bifidobacterium Breve Soluble Factors

**DOI:** 10.1371/journal.pone.0005184

**Published:** 2009-04-17

**Authors:** Elise Heuvelin, Corinne Lebreton, Corinne Grangette, Bruno Pot, Nadine Cerf-Bensussan, Martine Heyman

**Affiliations:** 1 INSERM U793, Paris, France; 2 Université Paris Descartes, Faculté de Médecine René Descartes, IFR94, Paris, France; 3 Laboratoire des Bactéries Lactiques et Immunité des Muqueuses, Institut Pasteur de Lille, IFR 142, Lille, France; Charité-Universitätsmedizin Berlin, Germany

## Abstract

**Objectives:**

Soluble factors released by *Bifidobacterium breve* C50 (*Bb*) alleviate the secretion of pro-inflammatory cytokines by immune cells, but their effect on intestinal epithelium remains elusive. To decipher the mechanisms accounting for the cross-talk between bacteria/soluble factors and intestinal epithelium, we measured the capacity of the bacteria, its conditioned medium (*Bb*-CM) and other Gram(+) commensal bacteria to dampen inflammatory chemokine secretion.

**Methods:**

TNFα-induced chemokine (CXCL8) secretion and alteration of NF-κB and AP-1 signalling pathways by *Bb* were studied by EMSA, confocal microscopy and western blotting. Anti-inflammatory capacity was also tested *in vivo* in a model of TNBS-induced colitis in mice.

**Results:**

*Bb* and *Bb-CM*, but not other commensal bacteria, induced a time and dose-dependent inhibition of CXCL8 secretion by epithelial cells driven by both AP-1 and NF-κB transcription pathways and implying decreased phosphorylation of p38-MAPK and IκB-α molecules. In TNBS-induced colitis in mice, *Bb*-CM decreased the colitis score and inflammatory cytokine expression, an effect reproduced by dendritic cell conditioning with *Bb*-CM.

**Conclusions:**

*Bb* and secreted soluble factors contribute positively to intestinal homeostasis by attenuating chemokine production. The results indicate that *Bb* down regulate inflammation at the epithelial level by inhibiting phosphorylations involved in inflammatory processes and by protective conditioning of dendritic cells.

## Introduction

Intestinal homeostasis depends in part on the equilibrium between absorption (nutrients, Na^+^ ions), secretion (chloride ions, IgA, mucus, cytokines) and barrier function of the digestive epithelium. Disturbance of intestinal homeostasis results in chronic inflammation and release of pro-inflammatory cytokines resulting in diarrhoea and injury to the gut. The intestinal epithelium constitutes a major interface between intestinal contents, including bacteria, and the internal milieu, providing an important contribution to the regulation of intestinal homeostasis. Probiotics are specific bacterial strains capable of stimulating protective immune responses in physiological conditions[1] but which also dampen inflammation in some inflammatory bowel diseases [2].

One of the main functions of the intestinal epithelium is to regulate the ion transport controlling the balance between absorption and secretion of fluid. Intestinal epithelial cells (IECs) are considered an integral part of the innate immune system, constituting important targets for bacteria and cytokines. Their polarization contributes to the fine regulation of intestinal homeostasis [3]–[5]. In the physiological steady-state, minimal stimulation of IECs by luminal bacteria occurs at the apical pole. Indeed, pathogen recognition receptors (PRRs) are functionally silenced in the healthy intestine due to their redistribution to internal or basolateral compartments or to the delivery of inhibitory signals [6], [7]. In contrast, in pathology, recognition of invading bacteria promotes signalling cascades of pro-inflammatory cytokines and chemokines[8] and the recruitment/activation of mucosal immune cells. In this context, selected strains of probiotic bacteria have been proposed as tools in the prevention or treatment of inflammatory bowel diseases, especially in ulcerative colitis [2]. Mechanisms sustaining such beneficial effects have been partially identified *in vitro* [9], [10] and depend mainly upon alleviation of NF-κB-dependent transcriptional activity. We have already demonstrated that soluble factors released by the probiotic bacteria *Bifidobacterium breve* (*Bb*) *C50* can down-regulate the production of inflammatory cytokines by immune cells [11], with these factors maintaining their inhibitory activity after crossing an epithelial barrier. Herein, we demonstrated the inhibitory effect of *Bb* soluble factors on epithelial signalling pathways leading to chemokine secretion in inflammation and confirmed their alleviating effect in a mouse model of colitis.

## Methods

### Bacteria and conditioned media

*Bifidobacterium breve* (*Bb*) *C50* was purchased from Bledina-SA (Steenvoorde, France). *Bifidobacterium breve 15698* and *Lactobacillus rhamnosus 10893* were purchased from ATCC, and *Eubacterium rectale L15*, isolated from a dominant human microflora, was provided by G. Corthier (INRA, Jouy en Josas, France). Bacteria cultured in BH medium were seeded for 24 hours in DMEM (Gibco) containing 10% fetal calf serum (FCS) and 2% inulin. *Bb*-conditioned-media corresponding to 3×10^8^ CFU/ml were prepared by ultracentrifugation (100,000 *g*, 1 h) and adjusted to pH 7.0. As demonstrated with immune cells, active soluble factors in *Bb*-conditioned media are less than 3 kD [11]. Conditioned media were thus ultrafiltrated on Centriplus® YM-3 (Millipore) and sterilized. This fraction constituted the *Bb*-conditioned medium (*Bb*-CM), also noted as “soluble factors” in this study.

### Effect of *Bb*, *Bb*-CM or commensal bacteria on chemokine secretion pathways in intestinal epithelial cells

HT29-19A cells were seeded on 24-well plates (Falcon®) at 2×10^5^ cells/well for 3 days. After overnight starving of FCS, monolayers were stimulated either basolaterally with TNFα (10 ng/ml) and/or apically with *Bb*-CM or *Bb* at increasing multiplicity of infection (MOI: 10, 50 or 100), i.e. number of bacteria per epithelial cell. Conditioned media were collected after 4 hours for cytokine assays.

### Human cytokine antibody array and CXCL8 assay

RayBio® Human Cytokine Antibody Array V membranes (Raybiotech Inc) were incubated with HT29-19A conditioned media and processed according to the manufacturer's instructions (Scheme of the various cytokines/chemokines detected can be found at http://www.raybiotech.com). Positive spots were analyzed with a CCD camera (Fuji LAS-1000 plus) and semi-quantified with the Image Gauge software (Molecular Dynamics).

Secretion of CXCL8 was assayed using the Duoset enzyme-linked immunosorbent assay (ELISA) (R&D Systems).

### Epithelial cell viability

As indexes of epithelial viability, we measured transepithelial electrical resistance (R), early apoptosis and zonula occludens 1 (ZO-1) distribution.

Filter-grown HT29-19A cell monolayers were cut-out from the insert and mounted in Ussing chambers. Potential difference (PD) and short-circuit current (I_sc_) were recorded and R was calculated according the ohm's law.

Epithelial cell apoptosis was assessed with the monoclonal antibody M30 CytoDEATH (Roche Diagnostics, Meylan, France) which recognizes cleaved cytokeratin 18, a marker of early apoptosis. A 10 min-treatment of epithelial cells with H_2_O_2_ (100 µM) was used as a positive control. Filter-grown HT29-19A cell monolayers (Costar® clear 3460) were incubated for 4 hours with *Bb*, and treated according to the manufacturer. Cells were incubated successively with mouse monoclonal IgG_2b_ anti-M30 (1∶10) and rabbit polyclonal anti-ZO-1 (24 µg/ml, Zymed, Clinisciences) for 60 and 30 min respectively. After washes, Cy5-goat anti-mouse IgG (H+L) and FITC-goat anti-rabbit (Jackson Laboratories, Immunotech, 15 µg/ml) were added for 30 min. Cell monolayers were observed by confocal microscopy.

### Activation of NF-κB and AP-1

#### Immunofluorescence analysis of nuclear translocation of p65-NF-κB

HT29-19A cells were grown for 3 days in eight-chamber slides (Lab-tek, Nunc), starved of FCS overnight and treated for 30 min with TNFα with or without *Bb*-CM. After fixation in formaldehyde 4%, cells were incubated for 90 min with a mouse monoclonal anti-p65 antibody (2 µg/ml, Santa Cruz) and FITC-goat anti-mouse IgG (Jackson laboratories) for 30 min. Nuclei were labelled using TOPRO-3 (0.04 µg/ml) and cell preparations observed using confocal microscopy.

#### Electrophoretic Migration Shift Assay (EMSA)

HT29-19A monolayers were treated with TNFα±bacteria for 4 hours and nuclear extracts (3 µg) were prepared and incubated in binding buffer with 0.35 pmol of ^32^P-labeled DNA probes corresponding to the κB or AP-1 binding sites (Promega) as previously described [12]. After 1 hour incubation, protein-DNA complexes were resolved in a 5% polyacrylamide gel in Tris-borate-EDTA buffer. The gel was exposed to a PhosphorImager screen (Molecular Dynamics) and quantified with ImageQuant software (Molecular Dynamics).

#### Western Blot analysis of p38 MAPK phosphorylation and IκB-α complex

HT29-19A cell lysates (30 µg) were fractionated on SDS-PAGE and transferred onto PVDF membrane. Membranes were blocked and incubated with mouse anti-phospho-p38 (2 µg/ml; Sigma-Aldrich), mouse anti-phospho-IκB-α (Ser32/36) (1∶200; Cell Signaling), rabbit polyclonal anti-p38 (12 µg/ml; Sigma-Aldrich), rabbit polyclonal anti IκB-α (1 µg/ml; Santa Cruz) or mouse anti-β-actin (2 µg/ml; Santa Cruz). Appropriate HRP-conjugated secondary antibodies were used and membranes were revealed with a CCD camera (Fuji LAS-1000 plus).

### Trinitrobenzene sulfonic acid (TNBS)-induced colitis in mice

Animal experiments were performed in an accredited establishment (n°A59107, animal facility of the Institut Pasteur de Lille, France) and carried out in accordance with the guidelines of laboratory animal care published by the French Ethical committee and the rules of the European Union Normatives (number 86/609/EEC). Colitis was induced by intra-rectal administration of 50 µl TNBS (110 mg/kg) in 0.9% (w/v) NaCl/ethanol (50∶50 v/v) in anesthetized BALB/c mice (female, 7–8-weeks-old) as described [13]. The colitis control group received TNBS only. The *Bb* i.g. group received a daily intragastric administration of 10^8^ CFU of *Bb* in 100 µl gavage buffer 5 days before colitis induction. The *Bb*-CM i.p. group received two intra-peritoneal (i.p. 200 µl) injections of a 5-fold concentrated *Bb*-CM at one day intervals before colitis induction. Other groups received a single i.p. administration of 2×10^6^ murine dendritic cells in 100 µl PBS, either untreated (DC group) or pre-conditioned with *Bb*-CM (DC*_Bb_*_-CM_ group) simultaneously to TNBS administration. DCs were prepared from bone marrow dendritic cells (BMDCs) as previously described [14] and incubated with or without *Bb*-CM for 18 hours.

Animals were sacrificed 48 h after TNBS administration and macroscopic colonic damage was analyzed using the Wallace scoring method [15]. Protection observed with the various treatments was calculated taking the Wallace score of TNBS mice (control) as the higest (100%) colonic damage. Total RNA was isolated from colon tissue using the RNeasy kit (Qiagen). Reverse transcription was performed on 2 µg, followed by real-time PCR (7300 PCR system from Applied Biosystems) using the Taq-Man PCR Master Mix (Applied Biosystems) and primers and probes designed by Applied Biosystems for murine IL-1β, TNF-α, CXCL1 (KC), COX-2, IL-6, IL-23 and IL-17 and TATA-box binding protein (Tbp). Data were normalized to expression of Tbp.

### Statistical analysis

Statistical analysis was performed using the SAS package. The results are expressed as scatter plots with medians, or as means±SEM. Differences between groups were compared by paired *t*-test or non-parametric tests (Wilcoxon) and were considered significant for values of p<0.05.

## Results

### *Bb* soluble factors inhibit secretion of inflammatory cytokines by intestinal epithelial cells

**Figure 1:**
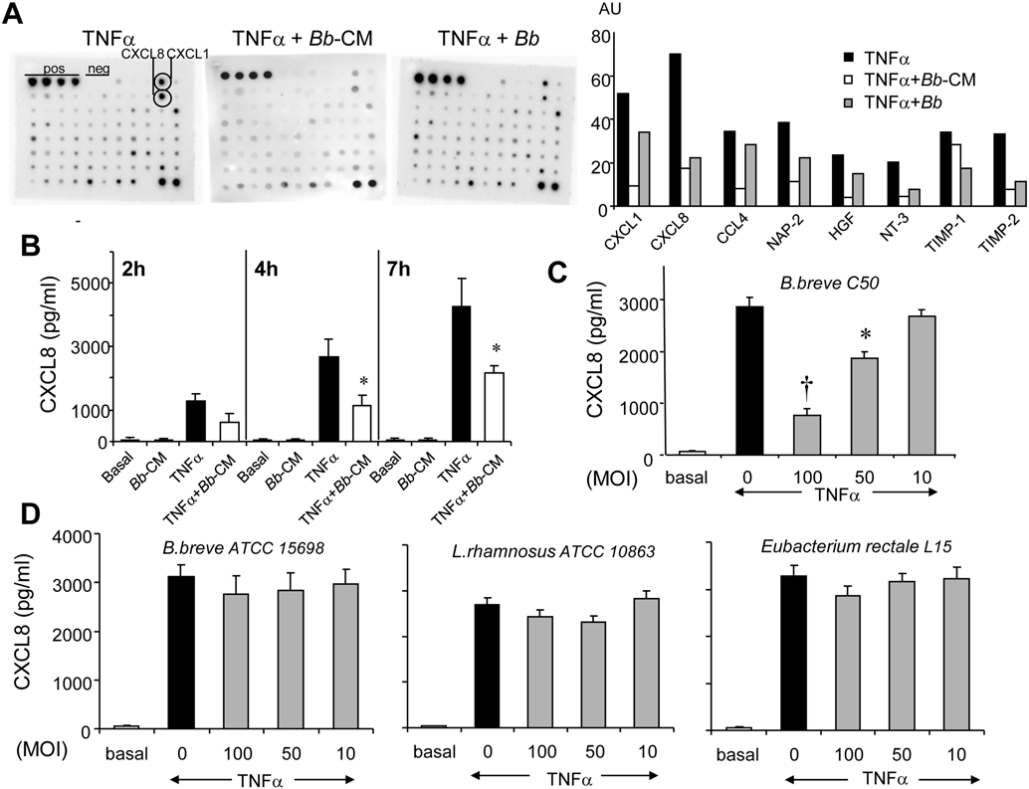
*Bb* and its soluble factors dampen TNFα-induced cytokine secretion in epithelial cells. A. After 4 hour-incubation of HT29-19A cells with TNFα±*Bb* (MOI 100) or *Bb*-CM, epithelial cell supernatants were tested by the Raybio® human cytokine array V (One representative array of two experiments). Secretion of chemokines (CXCL1, CXCL8, CCL4) and of other inflammatory molecules was inhibited in the presence of *Bb* and *Bb*-CM as judged by densitometric analysis. B. Long lasting inhibition (up to 7 hours) of CXCL8 secretion by *Bb*-CM (∼50%) was observed using ELISA. n = 7, *p<0.01 *vs* TNFα. C–D. HT29-19A cell monolayers were treated (4 hours) from the basolateral compartment with TNFα together with *Bb* or commensal bacteria (*B.breve* ATCC 15698, *L.rhamnosus* 10863, *E.rectale* L15) placed in the apical compartment at increasing MOI. As quantified by ELISA, *Bb* dose-dependently inhibited TNFα-induced CXCL8 secretion, in contrast to other bacteria. n = 11, †*p*<0.0001 ; *p<0.001 vs TNFα.

*Bb* soluble factors inhibit LPS-induced TNFα secretion in immune cells [11]. Their effect on TNFα-induced cytokine secretion in HT29-19A epithelial cells was tested using Raybio® membranes that allow screening of a large panel of inflammatory factors. *Bb* and *Bb*-CM inhibited TNFα-induced secretion of CXCL8 (IL-8), CXCL1 (GRO) and CCL4 (MIP-1β) and to a lesser extent of tissue inhibitor of metalloproteinases (TIMP-1, TIMP-2), neutrophil activating factor-2 (NAP-2), neurotrophin NT-3 and growth factors such as hepatocyte growth factor (HGF) (Fig. 1A). Importantly, *Bb* and *Bb*-CM alone did not modify the cytokine profile observed under basal conditions (data not shown). TNFα-induced secretion of CXCL8, tested by ELISA, increased with time but a constant fraction (50 to 58%) was inhibited by *Bb*-CM (1282.5±211 *vs* 611.5±282 pg/ml after 2 hours, 2695±555 *vs* 1134±342 pg/ml after 4 hours, p<0.01; and 4254±922 *vs* 2159±227 pg/ml after 7 hours; p<0.01) (Fig. 1B). Notably, *Bb*-CM also inhibited IL-1β-induced CXCL8 secretion (data not shown).

We next evaluated the capacity of apically applied *Bb* to inhibit TNFα-induced CXCL8 production in the basolateral compartment of polarized HT29-19A epithelial cells. Basal secretion of CXCL8 was not modified by *Bb* (data not shown), but *Bb* induced a dose-dependent inhibition of TNFα-induced CXCL8 secretion that was maximal at MOI 100 (73% inhibition, p<0.0001) (Fig. 1C). In contrast, no inhibition was observed with the other tested Gram (+) bacteria (Fig. 1D).

**Figure 2:**
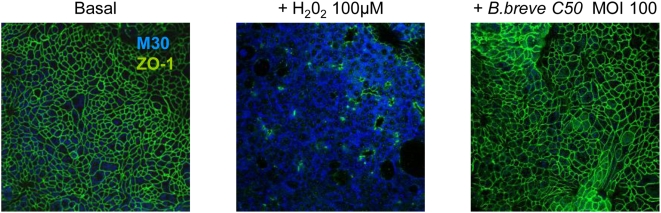
*Bb* preserves the integrity of HT29-19A epithelial cells. Filter-grown HT29-19A monolayers were treated with 10 ng/ml TNFα with or without live *Bb* placed in the apical compartment for 4 h. As index of epithelial viability, we measured markers of apoptosis (M30) and epithelial tight junction integrity (ZO-1). After 4 hour-treatment with *Bb*, HT29-19A monolayers (Costar® clear 3460) were labelled with the monoclonal antibody M30 CytoDEATH recognizing caspase-cleaved cytokeratin 18 (1∶10, Roche Diagnostics) and rabbit polyclonal anti-ZO-1 (24 µg/ml, Zymed). Cells were observed with the laser scanning confocal microscope LSM 510 Carl Zeiss. Treatment with H_2_O_2_ (100 µM), used as a positive control, induced apoptosis (blue M30 labeling) and alteration of ZO-1 distribution (green). In contrast, in basal condition or after treatment with *Bb* at MOI 100, no M30 labeling was observed and ZO-1 distribution was preserved. Results are representative of three independent experiments.

The inhibitory effects of *Bb* on chemokine secretion were not due to the alteration of HT29-19A epithelial integrity as attested by stable electrical resistance of epithelial monolayers in the presence of bacteria at high concentration (MOI 100, R = 98±11 ohms.cm^2^) as compared to control epithelial cells (R = 116±9 Ω.cm^2^). In addition, integrity of tight junctions was also attested by a normal distribution of ZO-1 protein and by the lack of bacteria-induced apoptosis of epithelial cells (Fig. 2).

### *Bb* soluble factors inhibit the NF-κB pathway

**Figure 3:**
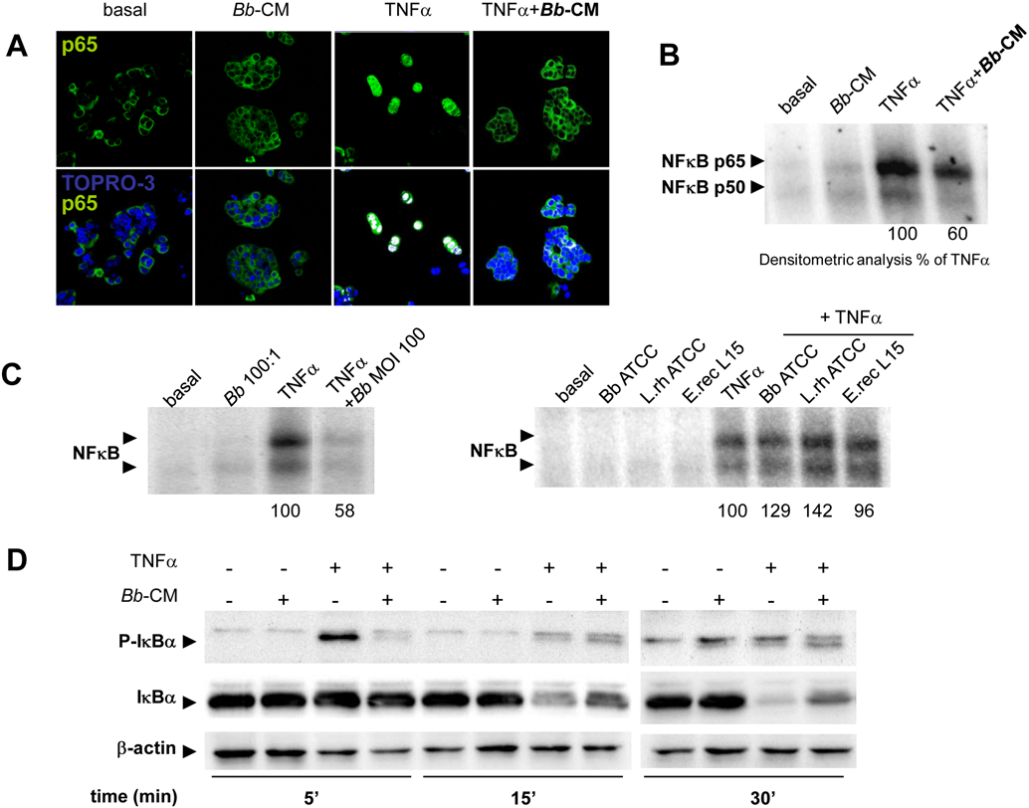
*Bb* and its soluble factors inhibit TNFα-induced NF-κB in epithelial cells. A. HT29-19A cells were incubated for 30 min with TNFα±*Bb*-CM and localization of the NF-κB p65 subunit was observed by confocal microscopy. Following exposure to TNFα, p65 (green) was translocated into the nuclei (blue) and co-localization was observed (white spots). *Bb*-CM inhibited TNFα-induced p65 subunit translocation. n = 3 independent experiments. B. HT29-19A cells were incubated for 4 hours with TNFα±*Bb*-CM. Electrophoretic migration shift assay (EMSA) was performed following extraction of nuclear proteins. TNFα condition was taken as 100% activation and *Bb*-CM induced a 40% inhibition of NF-κB activation (n = 2 independent experiments). C. Epithelial cells were incubated with TNFα±*Bb* at MOI 100. Activation of NF-κB was inhibited by ∼40% with *Bb* but not with commensal bacteria *B.breve* ATCC 15698, *L.rhamnosus* 10863, *E.rectale* L15. n = 2 independent experiments. D. HT29-19A cells were treated with TNFα±*Bb*-CM. Total proteins were separated by SDS-PAGE and revealed with antibodies to phospho-IκB-α, IκBα and β-actin. *Bb*-CM inhibited the TNFα-induced phosphorylation of IκB-α as early as 5 min and IκB-α degradation after 15 min. n = 3 independent experiments.

*Bb*-CM inhibited TNFα-induced nuclear translocation of the p65 NF-κB subunit. Nuclear translocation was observed in 83% of cells activated by TNFα alone, but in only 30% of cells preincubated with *Bb*-CM (Fig. 3A). Accordingly *Bb* soluble factors impaired the formation of p65- and p50-DNA complexes in response to a 4-hour stimulation with TNFα (Fig. 3B). In contrast to *Bb* (MOI 100), which inhibited by 40% the formation of the DNA complexes (Fig. 3C), other Gram (+) bacteria had no effect. Neither *Bb* nor other Gram (+) bacteria *per se* induced the binding of p65- and p50-NF-κB on epithelial DNA. As shown in Fig. 3D, *Bb*-CM also reduced the early phosphorylation of IκB-α (5 min) induced by TNFα − a step that otherwise commits the molecule to subsequent ubiquitination and degradation − and accordingly promoted stabilization of IκB-α from 15 to 30 min.

### *Bb* soluble factors inhibit the AP-1 pathway

**Figure 4:**
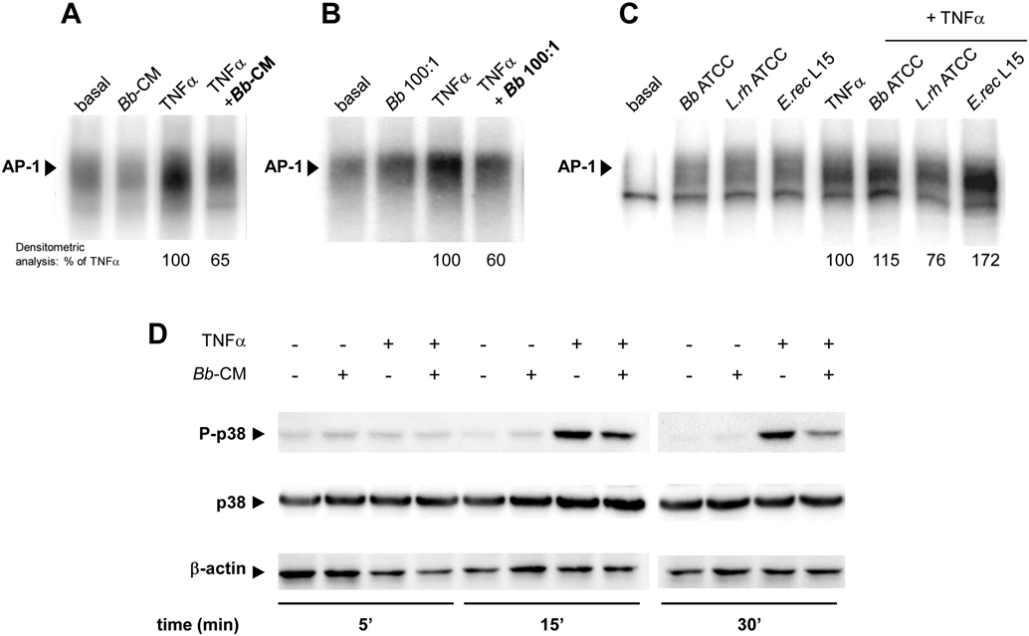
*Bb* and its soluble factors inhibit TNFα-induced phosphorylation of p38-MAPK in epithelial cells. A–C. HT29-19A cells were incubated for 4 hours on the apical surface with live *Bb* or commensal bacteria *B.breve* ATCC 15698, *L.rhamnosus* 10863, *E.rectale* L15 at MOI 100, together with basolateral TNFα. Activation of AP-1 assessed by EMSA was inhibited by ∼35% in the presence of *Bb*-CM and by ∼40% in the presence of *Bb*, but not in the presence of other bacteria. n = 2 independent experiments. D. HT29-19A cells were treated with TNFα±*Bb*-CM. Total proteins were analysed by SDS-PAGE and revealed with antibodies to phospho-p38-MAPK, p38-MAPK and β-actin. *Bb*-CM inhibited TNFα-induced phosphorylation of p38-MAPK from 15 to 30 min of incubation. n = 3 independent experiments.

DNA binding of AP-1 induced by TNFα was inhibited by 35% in the presence of *Bb*-CM (Fig. 4A) and by 40% by *Bb* at MOI 100 (Fig. 4B). In contrast to *Bb*, none of the tested Gram (+) bacteria had any inhibitory effect (Fig. 4C). Furthermore, *Bb*-CM inhibited p38-MAPK phosphorylation, an important step in AP-1 activation (Fig. 4D). This inhibition, observed at 15 min (- 44%), was still visible after 30 min (- 63%).

### *Bb* soluble factors alleviate inflammation in a murine model of TNBS colitis

**Figure 5:**
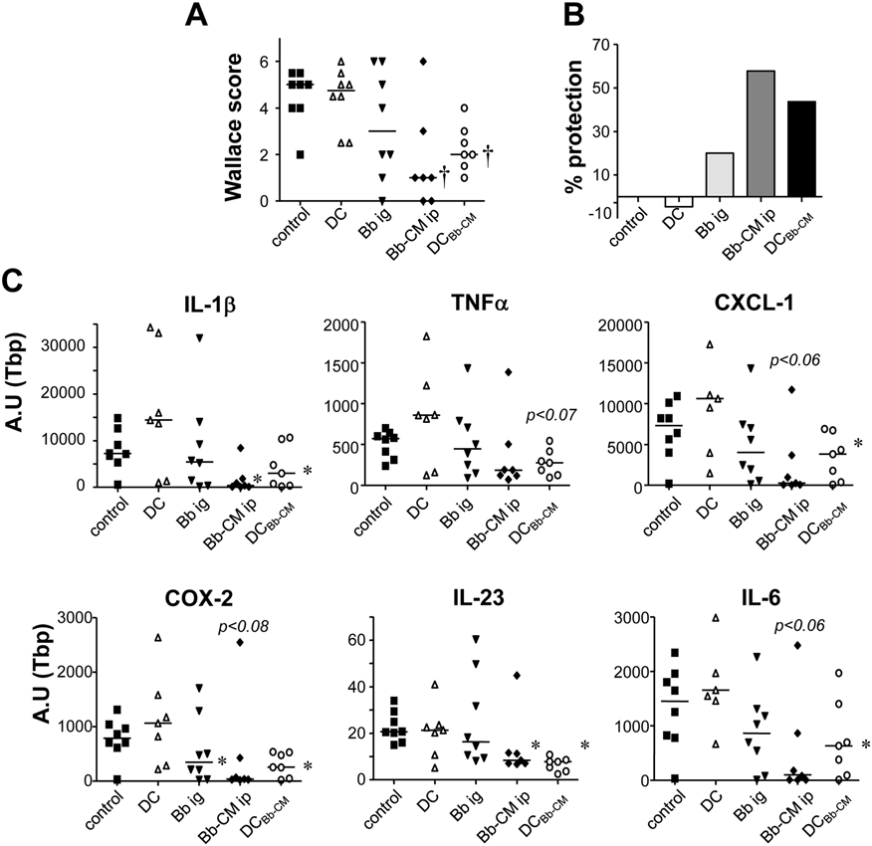
Protective effect of *Bb* and *Bb*-CM in TNBS-induced colitis in mice. A. Wallace scores were used to semi-quantify colitis activity in BALB/c mice after colonic instillation of TNBS (control group) with or without intragastric pre-treatment with 10^8^ CFU *Bb* (*Bb* i.g.), intraperitoneal injection of *Bb*-CM (*Bb*-CM i.p.), intraperitoneal injection of non treated-DC (DC) or *Bb*-CM conditioned-DC (DC*_Bb_*_-CM_). The mild inhibition of colitis observed with *Bb* i.g. became significant with *Bb-CM* and DC*_Bb_*_-CM_. B. Percentage protection in mice of the different groups compared to control (TNBS only). C. Quantitative RT-PCR analysis of mRNA expression of pro-inflammatory cytokines/mediators in the colon, 48 hours after TNBS-induced colitis with or without treatment. A significant inhibition of pro-inflammatory molecules in colonic mucosa (IL-1β, CXCL-1, IL-23, IL-6, COX2) was observed in the presence of *Bb*-CM or *Bb*-CM pre-conditioned DC. Scatter plots are presented with medians. *p<0.05, †*p*<0.01 *vs* TNBS; n = 7 to 8 mice per group.

We next investigated the capacity of *Bb* soluble factors to dampen the colonic inflammatory response in TNBS-induced colitis in mice. The high inflammatory score observed in control TNBS mice, (Wallace score WS = 4.5±0.4) was decreased in *Bb* i.g. mice (WS to 3.2±0.8), indicating a mild protection (30%) (Fig. 5A, 5B). Accordingly, high mRNA expression of pro-inflammatory cytokines/mediators observed in control TNBS mice slightly decreased in *Bb* i.g. mice (Fig. 5C). In contrast, pre-treatment with *Bb*-CM by i.p. route induced a significant protection against colitis (WS = 1.7±0.8, 62% protection) and a statistically significant decrease in pro-inflammatory cytokine expression [IL-1β, CXCL1 (equivalent to human IL-8), COX2, IL-23, IL-6]. Since i.p. administration of *Bb*-CM was efficient in dampening inflammation, the effect of soluble factors on mucosal dendritic cells (DC) was tested. DC pre-conditioned with *Bb*-CM (DC*_Bb_*_-CM_) were injected i.p. into mice at the same time as TNBS challenge. In contrast to non-treated DC (WS = 4.4±0.4), DC*_Bb_*_-CM_ conferred a significant protection against colitis (WS = 2.3±0.4, 49% protection, p<0.01) and reduced the expression of all pro-inflammatory cytokines/mediators. These results indicate a protective effect of *Bb* soluble factors *in vivo* and suggest that this protective effect relies primarily on their capacity to condition regulatory dendritic cells.

## Discussion

Selected strains of probiotic micro-organisms participate in the control of intestinal homeostasis and help preventing intestinal disorders. In the intestinal microflora, *Bifidobacterium* species are generally considered beneficial to the host and various strains of bifidobacteria are used as probiotics. The mechanisms by which these bacteria can modulate the function of epithelial and immune cells remain incompletely elucidated. We have previously shown that soluble factors from *Bifidobacterium breve* C50 decrease pro-inflammatory cytokine secretion by immune cells [11]. We now show that these soluble factors effectively dampen intestinal inflammation by targeting both epithelial and local dendritic cells.

In inflammatory conditions, an important function of intestinal epithelial cells is to control the influx and activation of immune cells into the *lamina propria* through the production of chemokines and cytokines. Previous studies have supported the view that selected commensal or probiotic bacteria can alleviate inflammation [16]. Along this line, we have already demonstrated that the low molecular weight soluble factors (<3 kD) produced by *Bb* inhibit LPS-induced TNFα secretion by immune cells [11]. In this study, *Bb* and *Bb*-CM, unlike other tested Gram (+) bacteria, were able to inhibit the release of chemokines and various inflammatory molecules in epithelial cells. The most prominent effect of *Bb*-CM was observed on CXCL8 secretion. Inhibition of CXCL8 secretion by probiotics has been reported in epithelial cells but the relative importance of whole bacteria *versus* soluble factors have remained elusive [17], [18]. Different steps along the NF-κB pathway were targeted according to bacterial strains tested. Commensal *Salmonella* [9] or *Lactobacillus casei* [10] could inhibit p65 nuclear translocation through a decrease in IkB-α ubiquitination and degradation, *B. thetaiotaomicron* commensal bacteria promoted the nuclear export of RelA and PPAR-γ[19] while VSL#3 inhibited proteasome degrading activity[20] without altering IkB-α phosphorylation. Our study indicates that both pathways implicated in CXCL8 expression, NF-κB and AP-1 [21], are targeted by *Bb* soluble factors. Interestingly, *Bb* inhibited key early phosphorylation steps in these two distinct signaling cascades. These results suggest that as yet unknown soluble factor(s) present in the low molecular weight active fraction of *Bb*-CM interfere with serine-threonine kinases and cellular phosphorylation. The nature of this(ese) factor(s) is not yet elucidated. Previous studies [11] underlined that inhibition of LPS-induced TNFα-release in immune cells by *Bb* C50 was heat-resistant, was not related to short-chain fatty acids (butyrate or lactate) and that pepsin-trypsin- or proteinase K- treated CM still retained an inhibitory effect ([11] and unpublished result). Additional unpublished studies indicated that DNAse I treatment of CM or the use of immune cells from TLR9−/− mice[22] did not point to the role of TLR9 ligands as active component(s) of *Bb*-CM. Studies aiming to characterize the active factors by differential mass spectrometry of active and inactive Bifidobacterium strains (*Bb* C50 and *Bb* ATCC 15698) are in progress and may help gaining further mechanistic insight.

To assess whether *in vitro* observations could be validated *in vivo*, we used a mouse model of colitis. Significant protection was observed after pre-treatment by i.p. injection of *Bb* soluble factors. Similar protection was obtained after adoptive transfer of dendritic cells pre-treated with *Bb*-conditioned medium. These results are coherent with our previous *in vitro* results in immune cells[11] and indicate that *Bb*-derived soluble factors can exert *in vivo* anti-inflammatory effects through interaction with local immune cells, as also reported with selected strains of lactic acid bacteria [14]. When *Bb* was administered intragastrically, a milder protection was observed, suggesting that the anti-inflammatory properties of *Bb* require the release of sufficient amounts of soluble factors *in situ*, in the vicinity of colonic mucosa. In our previous study, the inhibitory capacity of soluble factors on immune cells was maintained after crossing an epithelial cell monolayer. However the stability of these factors in the intestinal lumen may be insufficient to allow their delivery to the colonic mucosa. This hypothesis is in keeping with the observation that protection by probiotics is often more effective in the proximal intestine (rotavirus diarrhea) than in the colon (inflammatory bowel diseases). In this respect, it is interesting that *Fecalibacterium prausnitzii*, a commensal strain poorly represented in the microbiota of patients with Crohn's disease, can produce soluble anti-inflammatory factors which may thus be delivered directly to the adjacent mucosa [23].

Taken together, our results indicate that small soluble factors released by *Bifidobacterium breve* C50 might help maintain intestinal homeostasis by targeting cells of the innate immune system such as epithelial and dendritic cells. Studies are needed to further characterize the *Bb* soluble factor(s) responsible for the inhibition of kinases involved in multiple steps of intestinal inflammation.
